# Rapid Genotyping of β-tubulin Polymorphisms in *Trichuris trichiura* and *Ascaris lumbricoides*

**DOI:** 10.1371/journal.pntd.0005205

**Published:** 2017-01-12

**Authors:** Nour Rashwan, Marilyn Scott, Roger Prichard

**Affiliations:** Institute of Parasitology, Macdonald College, McGill University, Ste Anne de Bellevue, Quebec, Canada; Swiss Tropical and Public Health Institute, SWITZERLAND

## Abstract

**Background:**

The benzimidazole (BZ) anthelmintics, albendazole (ABZ) and mebendazole (MBZ) are the most common drugs used for treatment of soil-transmitted helminths (STHs). Their intensive use increases the possibility that BZ resistance may develop. In veterinary nematodes, BZ resistance is caused by a single nucleotide polymorphism (SNP) in the β-tubulin isotype 1 gene at codon position 200, 167 or 198, and these SNPs have also been correlated with poor response of human *Trichuris trichiura* to BZ treatment. It is important to be able to investigate the presence of resistance-associated SNPs in STHs before resistance becomes clinically established.

**Methods:**

The objective of this study was to develop new genotyping assays to screen for the presence of β-tubulin SNPs in *T*. *trichiura* and *Ascaris lumbricoides*. Rapid, simple and accurate genotyping assays were developed based on the SmartAmp2 method. Primer sets were optimized and selected to distinguish the SNP-variant genotypes. After initial optimization on control plasmids, the feasibility of the assay was assessed in field samples from Haiti and Panama. Finally, spiked fecal samples were assessed to determine the tolerance of *Aac* polymerase to fecal inhibitors.

**Findings:**

Rapid SNP genotyping assays were developed to target β-tubulin polymorphisms in *T*. *trichiura* and *A*. *lumbricoides*. The assays showed high sensitivity and specificity in field samples and also demonstrated high tolerance to PCR inhibitors in fecal samples.

**Conclusion:**

These assays proved to be robust and efficient with the potential to be used as field tools for monitoring SNPs that could be associated with BZ resistance. However, further work is needed to validate the assays on large numbers of field samples before and after treatment.

## Author Summary

The soil-transmitted helminths (STHs) *Ascaris lumbricoides*, *Trichuris trichiura*, and the hookworms *Necator americanus* and *Ancylostoma duodenale*, are the most prevalent intestinal helminths of humans, causing morbidity in developing countries. Large-scale preventive chemotherapy with albendazole or mebendazole is the major control strategy against STHs in mass drug administration (MDA) programs. In veterinary parasites, intensive reliance on the same anthelmintics has led to the emergence of benzimidazole (BZ) resistance, which is caused by a single nucleotide polymorphism at either codon 200, 167 or 198 in the β-tubulin isotype 1 gene. A great concern is that the intensive use of anthelmintics with suboptimal efficacy may lead to the development of resistance in human STHs. We developed novel genotyping assays to screen for β-tubulin polymorphisms in *T*. *trichiura* and *A*. *lumbricoides* based on the SmartAmp2 method. SmartAmp2 is a unique genotyping technology that detects a mutation under isothermal conditions with high specificity and sensitivity. The SNP detection assays were rapid, sensitive and highly specific for the detection of SNPs associated with BZ resistance with the potential to be used for monitoring in the field.

## Introduction

Soil-transmitted helminths (STHs) are a major cause of morbidity in developing countries. *Ascaris lumbricoides*, *Trichuris trichiura* and the hookworms *Necator americanus* and *Ancylostoma duodenale* are estimated to infect more than 1.5 billion people, resulting in approximately 5.2 million disability adjusted life years (DALYs) lost worldwide [1, 2]. Pre-school and school-age children are the most at risk of heavy infection with STHs and of developing severe morbidity [3, 4], leading to malnourishment, stunted growth and intellectual retardation, with cognitive and educational deficits [5]. Recent estimates indicate that approximately 900 million children are at high risk of acquiring STH infection and in need of annual treatment [6].

The current control strategy against STHs is the regular administration of a single-dose of ABZ (400 mg) or MBZ (500 mg) as preventive chemotherapy in large-scale mass drug administration (MDA) programs [7] with the ultimate goal of elimination of STHs as a public health problem by 2020 [3]. These programs have been greatly expanded in recent years by massive donations of these drugs. A single-dose of ABZ or MBZ shows high efficacy against *A*. *lumbricoides*, but both drugs show significantly suboptimal efficacy against *T*. *trichiura* and hookworm [8–11]. Intensive and prolonged reliance on two drugs of the same anthelmintic class with the same mode of action and suboptimal efficacy greatly increases the probability that BZ resistance may develop [12–14]. This would raise serious complications for control of STHs [4].

In veterinary nematodes, resistance developed in response to heavy reliance for many years on BZ anthelmintics [13]. It was found that the BZ resistance is caused by a single nucleotide polymorphism (SNP) in the β-tubulin isotype 1 gene at codon 167, codon 200 (TTC>TAC) or at codon 198 (GAG>GCG) [15–18]. Such SNPs have already been observed in *T*. *trichiura* and *N*. *americanus* [19,20]. Additionally, the frequency of SNPs at codon 200 and 198 increased with treatment and was significantly higher in individuals who showed a poor response to ABZ than in individuals who responded well to ABZ in *T*. *trichiura* [21]. To maintain the benefits of MDA programs, it is important to have tools that can be used for large-scale screening for BZ resistance in human STHs. The lack of detection of phenotypic resistance may, in part, be due to the lack of a reliable and sensitive method to monitor for resistance genotypes before and after BZ treatment [22], a low frequency of resistance alleles, and the probability that BZ resistance is recessive, as it is in veterinary parasites [23].

PCR-based methods such as real-time PCR (RT-PCR) and pyrosequencing have been developed and applied for the detection of putative BZ resistance SNPs in human STH [19, 20, 24]. Diagnostic RT-PCR is a rapid detection method in which primers bind only to specific sequence variants, with the 3’-end overlapping the SNP of interest. Allele-specific RT-PCR was developed for monitoring for β-tubulin polymorphisms in the human hookworms *A*. *duodenale* and *N*. *americanus* [24]; however, this method lacks the capability to completely distinguish background amplification noise rising from a non-target sequence [25]. Pyrosequencing has been developed for detection of resistance-associated SNPs in many veterinary parasites [26, 27] and also in human parasites [19, 20, 28]. Compared with RT-PCR, pyrosequencing is quicker and easier to perform as it allows testing multiple SNPs. However, the equipment required is expensive and not widely available [27]. Additionally, careful DNA purification is needed as the *Taq* DNA polymerases can be inhibited by impurities in clinical samples [29]. Intolerance to fecal inhibitors could result in a high percentage of PCR failure, which in turn, limits the ability to identify polymorphisms and to draw a conclusion, particularly in samples that have low DNA concentration. Current PCR-based methods have been shown to be more accurate, sensitive, and convenient than conventional sequencing; however, they are still time consuming procedures, require careful DNA extraction and expensive equipment, and include multiple steps. Therefore, they are not suitable for large scale screening and are difficult to implement.

Here we report the development of a novel genotyping assay to monitor the presence or absence of these SNPs in the β-tubulin isotype 1 gene of *T*. *trichiura and A*. *lumbricoides*, using the SmartAmp2 method (Smart Amplification Process). This technique has been previously used to develop assays for the hookworm, *N*. *americanus* [30]. The SmartAmp2 is a DNA amplification method for detection of DNA mutations, deletions or SNPs under isothermal conditions and in a single step. This method detects a single nucleotide polymorphism with high specificity and sensitivity within 30–45 min [25, 31]. SmartAmp2 uses *Aac* DNA polymerase (a strand displacing polymerase) combined with asymmetric primer design and *Taq* MutS *(Thermaus aquaticus* MutS) enzyme, which promote high specificity [32, 33].

The aim of this study was therefore to develop rapid and accurate genotyping assays based on the SmartAmp2 method for the detection of the β-tubulin SNPs that are likely associated with BZ resistance in *T*. *trichiura* and *A*. *lumbricoides*, and to validate their specificity and reliability in field samples.

## Materials and Methods

### Study approval and ethical considerations

Ethical approval for samples from Panama was obtained from the McGill University Institutional Review Board approval number A09-M87-11A [34, 35]. A parent or legal guardian gave informed consent for every child providing a stool sample.

Ethical approval (study 2535) was obtained by Dr. Patrick Lammie, CDC, Atlanta, GA, and included the collection, examination of fecal samples from Haiti, for helminth eggs, and DNA analysis of helminth eggs. Oral informed consent was obtained from all human adult participants and from parents or legal guardians of minors, as described previously (20, 21). Based on experience, it was considered unlikely that most persons in the sample communities would be literate. A request was therefore made to the Institutional Review Board for a waiver of written documentation of informed consent on the basis that the research presented no more than minimal risk of harm to the subjects and involves no procedures for which written consent is normally required outside of the research context in this setting. The reader of the consent form and a witness were asked to sign the form to indicate the subject’s agreement. The procedure was approved by the Institutional Review Board. This approach had been successfully used as part of recent studies and the forms were based on Institutional Review Board approved protocols (CDC Protocol #1524). Subjects were offered a written copy of the consent form.

### Parasite materials and DNA extraction

*A*. *lumbricoides* eggs and adult worms had been collected as previously described [19]. *T*. *trichiura* DNA from adult worms was donated by Dr. P. Nesjum, University of Copenhagen, Denmark [36], and 40 fecal samples were collected in Haiti from children that were naturally infected. From Panama, 34 fecal samples were collected. All samples were preserved in 70% ethanol after collection. Eggs were isolated under a dissecting microscope using a 10 μl pipette and genomic DNA was extracted from eggs as described [37]. Lysis buffer was prepared as follow (KCl [50 mM], Tris[10 mM] pH 8.3, MgCl_2_ [2.5 mM], 0.45% Nonidet P-40, 0.45% Tween 20 and 0.01% gelatine). Ten μl of proteinase-K [10 μg/ml] (Invitrogen, Life Technologies; Burlington, ON) and β-mercaptoethanol (Sigma-Aldrich, ON, Canada) were added to 1 ml of this buffer, just before use. Twenty-five μl of the resulting lysis buffer mix was added to previously isolated eggs and then tubes were incubated at 60°C for 2 h. Genomic DNA was extracted from adult *A*. *lumbricoides* using DNeasy tissue extraction kit (Qiagen, Mississauga, ON, Canada) according to the manufacture’s protocol.

### Wild-type and mutant-type plasmid constructs

To assist with SmartAmp2 development, wild-type (WT) and mutant-type (MT) plasmids were engineered and used as DNA templates for assay optimization. Extracted genomic DNA from individual adult worms was used to amplify a 472 bp fragment of the *T*. *trichiura* β-tubulin isotype 1 gene, including codon positions 167, 198 and 200. Specific forward primer 5’-GGCTAAAGGGCACTATACG-3’ and specific reverse primer 5’-GGAAAGCGTAGGCATGTCG-3’ (Invitrogen) were designed in the exonic regions of *T*. *trichiura* genomic DNA sequence (GenBank accession. no. AF034219.1). Genomic DNA extracted from *A*. *lumbricoides* adult worms was used to amplify a 564 pb fragment of the *Ascaris* β-tubulin isotype 1 gene, including codon positions 167, 198 and 200. Specific forward primer 5’- CCAGCTGACGCACTCGCTTGG -3’ and Specific reverse primer 5’-ATGGTTGAGGTCTCCGTATGTG-3’ (Invitrogen) were designed on the mRNA sequence of *A*. *lumbricoides* (GenBank accession. no. EU814697.1).

The PCR master mix contained 2 μl 10×PCR buffer, 1 μl MgSO_4_ [50 mM], 1 μl dNTP [10mM], 1 μl forward and reverse primer [10μM], 1 U Platinum *Taq* DNA polymerase High Fidelity (Invitrogen), 2 μl genomic DNA and distilled H_2_O to reach a final volume of 20 μl. No-template controls were also included for quality control. The PCR reaction conditions were the same for the two species (94°C for 3 min, followed by 35 cycles at 94°C for 45 s, 59°C for 45 s and 68°C for 1 min and a final extension at 68°C for 10 min. The resulting PCR fragments were Sanger sequenced to confirm the presence of WT alleles at codon positions 167, 198, and 200. MT plasmids carrying mutations at position 167, 198, or 200 were engineered by site-directed mutagenesis. *A*. *lumbricoides* and *T*. *trichiura* primers for MT plasmid construction, including inner primers carrying the mutant alleles and outer primers, are shown in Tables 1 and 2. WT and MT amplified fragments were cloned into TOPO-TA-Cloning vector (Invitrogen). Plasmid DNAs were extracted and purified using QIAprep Spin Miniprep kit (Qiagen) and subsequently sequenced by Sanger sequencing at the McGill University/Genome Quebec Innovation Centre, Montreal, Quebec. The purity and quantity of DNA in clones was measured using a Nano Drop photometer (Implen, Munich, Germany). WT and MT plasmids were used for assay optimization and development.

**Table 1:** *Trichuris trichiura* specific primers for the mutant plasmid constructs.

Codon	Primer sequences (5′–3′)
198	**Forward**: GGCTAAAGGGCACTATACG
	**Reverse**: GGAAAGCGTAGGCATGTCG
	**SNP Fwd**: GTAGAGAACACGGACG**C**AAC
	**SNP Rev**: GTT**G**CGTCCGTGTTCTCTAC
200	**Forward**: GGCTAAAGGGCACTATACG
	**Reverse**: GGAAAGCGTAGGCATGTCG
	**SNP Fwd**: GAACACGGACGAAACAT**A**CTG
	**SNP Rev**: CAG**T**ATGTTTCGTCCGTGTTC

**Table 2:** *Ascaris lumbricoides* specific primers for the mutant plasmid constructs.

Codon	Primer sequences (5′–3′)
167	**Forward**: CCAGCTGACGCACTCGCTTGG
	**Reverse**: GGTTGAGGTCTCCGTATGTG
	**SNP Fwd**: GCTCGT**A**CTCAGTTGTTCCATC
	**SNP Rev**: GATGGAACAACTGAG**T**ACGAGC
198	**Forward**: CCAGCTGACGCACTCGCTTGG
	**Reverse**: GGTTGAGGTCTCCGTATGTG
	**SNP Fwd**: GAACACCGATG**C**AACCTTC
	**SNP Rev**: GAAGGTT**G**CATCGGTGTTC
200	**Forward**: CCAGCTGACGCACTCGCTTGG
	**Reverse**: GGTTGAGGTCTCCGTATGTG
	**SNP Fwd**: GAACACCGATGAAACCT**A**CTG
	**SNP Rev**: CAG**T**AGGTTTCATCGGTGTTC

* SNP-Fwd: forward primer mutated for a single nucleotide; SNP-Rev: reverse primer mutated for a single nucleotide.

### SmartAmp2 primer design

**Fig 1:**
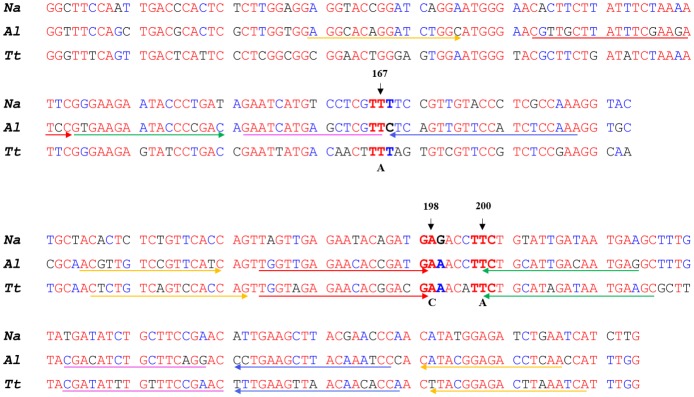
Partial sequence alignment of β-tubulin isotype 1 gene for *Necator americanus*, *Ascaris lumbricoides* and *Trichuris trichiura*. SNP 167T>A, SNP 198A>C, and SNP 200T>A SmartAmp2 primer sets were designed with the 3’-end or 5’-end of one or two primers are overlapping inter-species nucleotide variations (species-specific).

**Fig 2:**
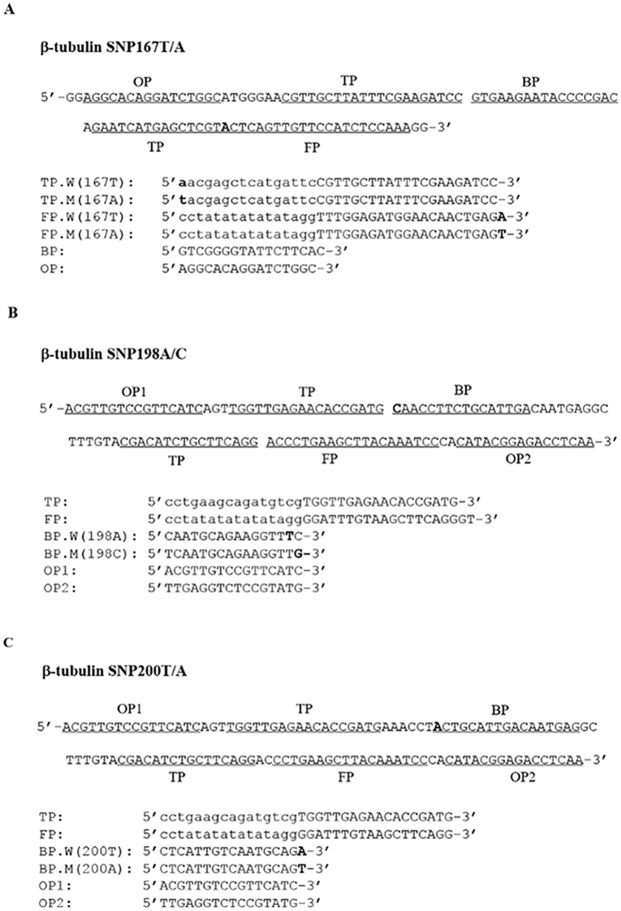
SmartAmp2 primer design for β-tubulin SNP detection in *Ascaris lumbricoides*. Partial sequence of the β-tubulin isotype 1 gene carrying (**A**) SNP 167A, (**B**) SNP 198C, and (**C**) SNP 200A as well as the sequences of primers used for the SmartAmp2 assay for the three SNP positions. The locations of SNPs indicated in bold. Both TP (turn-back primer) and FP (folding primer) were used as discrimination primers to target F167Y (T**T**C>T**A**C) SNP. BP (boost primer) was used as discrimination primer to target E198A (G**A**A>G**C**A) and F200Y (T**T**C>T**A**C) SNPs. The folding primer (FP) has a specific sequence (CCTATATATATATAGG) at the 5’ end to allow self-annealing hairpin formation.

**Fig 3:**
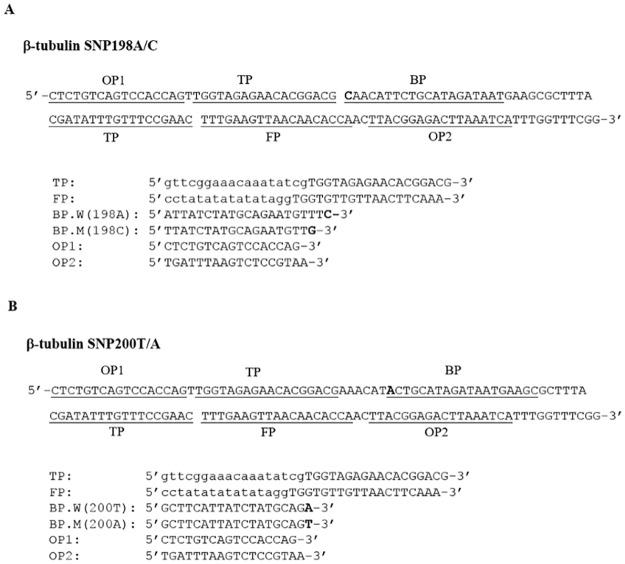
SmartAmp2 primer design for β-tubulin SNP detection in *Trichuris trichiura*. Partial sequence of the β-tubulin isotype 1 gene carrying (**A**) SNP 198C (**B**) SNP 200A as well as the sequences of primers used for the SmartAmp2 assay for the two SNP positions. The locations of SNPs indicated in bold. BP (boost primer) was used as discrimination primer to target E198A (G**A**A>G**C**A) and F200Y (T**T**C>T**A**C) SNPs. The folding primer (FP) has a specific sequence (CCTATATATATATAGG) at the 5’ end to allow self-annealing hairpin formation.

Primer sets were designed specifically to amplify and detect the F167Y (T**T**C>T**A**C), F200Y (T**T**C>T**A**C) and E198A (G**A**A>G**C**A) SNPs of the β-tubulin isotype 1 gene of *A*. *lumbricoides* and F200Y (T**T**C>T**A**C) and E198A (G**A**A>G**C**A) SNPs in the *T*. *trichiura* β-tubulin isotype 1 gene. The online software version 1.1 (www.SMAPDNA.com) was used initially to design several primer sets on the forward and reverse sequences with different discrimination primers which bind only to specific sequence variants, with the 3’-end overlapping the SNP of interest. Further refinements in the primer design were made by running evaluation tests and the best candidate primer sets were selected for each assay. A primer set consisting of five specific primers [the folding primer (FP), turn-back primer (TP), boost primer (BP), and two outer primers (OP1 and OP2)] were designed to recognize six different sequences on the target DNA. All primers were designed to be species-specific based on β-tubulin isotype 1 alignments (http://multalin.toulouse.inra.fr/multalin/) for *A*. *lumbricoides*, *T*. *trichiura* and *N*. *americanus* (Fig 1). The location and the sequences of primers for each SNP target are illustrated in Figs 2 and 3.

### SmartAmp2 assay development and optimization

Control plasmids encoding WT or MT alleles were used to develop each assay and to evaluate the accuracy of genotyping between different primer sets. Accordingly, a candidate primer set was developed and optimized to distinguish MT and WT genotypes. Further optimization of assay conditions and components (concentrations of primers, MgSO_4_, betaine, SYBR Green, polymerase and *Taq* MutS) was performed. SmartAmp2 reactions were carried out in a total volume of 25 μl containing 2 μM TP/FP, 1 μM BP, 0.25 μM OP1/OP2 (Invitrogen), 1.4 mM dNTPs (Invitrogen), 1 M betaine (Sigma- Aldrich), 1x isothermal buffer [20 mM Tris-HCl (pH 8.6), 10 mM KCl, 10 mM (NH_4_)_2_SO_4_, (4–8 mM) MgSO_4_, 0.1% Tween 20], 1/100,000 dilution SYBR Green I (Invitrogen), 1 μg *Taq* MutS (Wako Chemicals, USA /Nippon Gene CO, Japan), and 12 U *Aac* DNA polymerase (KK. DNAFORM, Japan). One microliter of each WT or MT plasmid (5 ng) was heated at 95°C for 5 min before being added to the assay. Reactions were incubated at 60°C for 60 min. The Rotor-Gene Q system (Qiagen) was used to maintain isothermal conditions and to monitor the change in fluorescence intensity of SYBR Green I during the reaction. Assays were evaluated in terms of full match amplification and mismatch (non-amplification) within 60 min.

### SmartAmp2 assay sensitivity and specificity

Following optimization of the assay with WT and MT plasmids, the specificity of each SNP-detection assay was verified with MT plasmids (F167Y, F200Y or E198A) of different STHs. The aim was to assess whether SNPs of another STH species would affect the specific amplification of each SNP-detection assay. Further optimization was performed to estimate the sensitivity and reproducibility of the assay. Extracted gDNA from individual adult worms, individual eggs and pools (10 eggs/pool) was tested. After DNA extraction from eggs using lysis buffer and proteinase K, 3μl of this crude lysate was added to the reactions and then tubes were incubated at 60°C for 90 min. Positive (WT and MT plasmids) and negative controls (no template) were included and the experiments were repeated twice, each time in duplicate.

### Genotyping of *T*. *trichiura* and *A*. *lumbricoides* field samples

Validation for field samples was performed with pools of *A*. *lumbricoides* and *T*. *trichiura* eggs obtained from Haiti and Panama. Pools of 10 eggs previously collected under microscopy for each stool sample were analyzed. Eggs were digested using 25 μl of previous lysis buffer mix. From this crude lysate, 3 μl were added to each reaction after a DNA heating step at 95°C for 3–5 min. Assays were carried out as previously described. Positive and negative controls were always included as references in each experiment. Tubes were incubated in a RT-PCR system at 60°C for 90 min.

### Assessment of SmartAmp2 polymerase tolerance to fecal inhibitors

To evaluate the tolerance of *Aac* polymerase to inhibitors in fecal samples, fecal samples that were confirmed to be negative for STH eggs were spiked with a known number of *T*. *trichiura* or *A*. *lumbricoides* eggs and then DNA was extracted according to the following modified protocol. Approximately 1 g of feces, preserved in 70% ethanol, was centrifuged and the fecal pellet was washed 3 times in PBS (phosphate buffered saline) and centrifuged. PBS solution was added to the fecal pellet to a final volume of 1 ml. Ten aliquots of 100 μl of fecal homogenate were transferred to new tubes. Tubes were centrifuged and excess PBS was removed. Each fecal sample was spiked with either *A*. *lumbricoides* or *T*. *trichiura* eggs (~10 eggs/tube). Other fecal samples were not spiked and were used as negative fecal controls.

DNA was extracted as follow: Fecal samples were frozen at -80°C for 30 min, and 10 μl of buffer A [NaOH (200 mM) + 2% Tween-20] was added to each tube. After a 15 min incubation period at 25°C, tubes were heated at 99°C for 10 min. Tubes were allowed to cool down, and then 10 μl of Buffer B [Tris-HCl (100 mM) and 2mM EDTA] were added and a second heat shock at 98°C for 2 min was performed. Finally, samples were centrifuged and the supernatants were transferred to a new PCR tube. Sample preparations were diluted 4-fold with distilled H_2_O, heated at 95°C for 3 min, cooled on ice and then 1 μl was added to the SmartAmp2 reaction mixture (total volume of 10 μl) containing 2 μM TP/FP, 1μM BP, 0.25 μM OP1/OP2 1.4 mM dNTPs, 1 M betaine, 1x isothermal buffer [20 mM Tris-HCl (pH 8.6), 10 mM KCl, 10 mM (NH_4_)_2_SO_4_, 8 mM MgSO_4_, 0.1% Tween 20], 2 μg bovine serum albumin (BSA) (Sigma- Aldrich,), 1/100,000 dilution SYBR Green I, 0.5 μg *Taq* MutS, and 5 U *Aac* DNA polymerase. BSA was added to the SmartAmp2 reaction mix to stabilise the DNA polymerase and to neutralise fecal inhibitors. Reactions were incubated at 60°C for 90 min in a RT-PCR system to maintain isothermal conditions and to monitor the change in fluorescence intensity of SYBR Green I during the reaction. Assays were evaluated in terms of full match amplification and mismatch amplification (non-amplification).

## Results

### SmartAmp2 primer design

Various sets of primers were designed to genotype F167Y (T**T**C>T**A**C), F200Y (T**T**C>T**A**C) and E198A (G**A**A>G**C**A) SNPs of the β-tubulin isotype 1 gene. Screening of these primer combinations under a variety of assay conditions identified an ideal primer set for each assay which achieved the best yield, speed and efficiency to discriminate between MT and WT genotypes. The location and sequences of primers are shown in Figs 2 and 3. For *A*. *lumbricoides*, a primer set was developed and optimized specifically to target the SNP F167Y (T**T**C>T**A**C) found in some *A*. *lumbricoides* samples [21]. Both the 5’-end of TPs and the 3’-end of FPs were employed to overlap and discriminate the F167Y SNP. Primer sets were optimized specifically to target the F200Y (T**T**C>T**A**C) or E198A (G**A**A>G**C**A) SNPs in *A*. *lumbricoides* and *T*. *trichiura*. The 3’-end of BPs were employed to discriminate the MT and the WT genotypes.

### SmartAmp2 assay optimization

**Fig 4:**
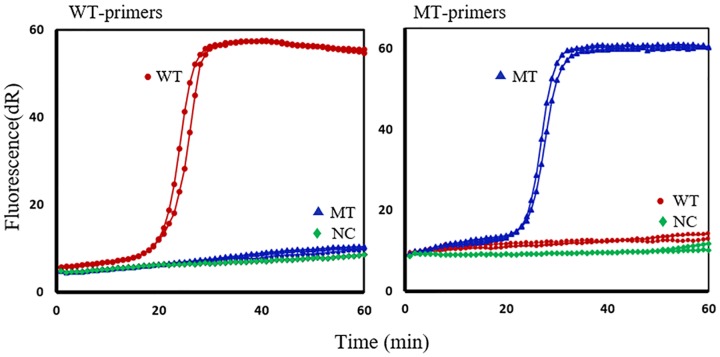
SmartAmp2 assay optimization for *Trichuris trichiura* β- tubulin polymorphisms. Left graph, wild-type (WT)-primer amplification (full match) of WT plasmid (red circle) with no amplification (mismatch) with mutant-type (MT) plasmid (blue triangle) or NC (no template) (green diamond). Right graph, MT-primer amplification of MT plasmid (blue triangle) with no amplification with WT plasmid (red circle) or NC (green diamond). All experiments were run in duplicates. dR, difference of relative fluorescence unit.

Sequencing of the WT and MT plasmids revealed that the desired mutations at codon positions 167, 198 and 200 of the β-tubulin gene were generated. WT and MT plasmids were used initially as DNA templates for assay optimization and development. Primer sets that exhibited delayed full match amplification or displayed a short delay between the full-match and mismatch amplification were omitted. Optimal amplification results were obtained when the reaction mixture contained 2 μM TP/FP, 1 μM BP and 0.25 μM OP1/OP2 with 1 M of betaine and 8 mM MgSO_4_. Mutant primer sets designed specifically to target the F167Y (T**T**C>T**A**C), F200Y (T**T**C>T**A**C) or E198A (G**A**A>G**C**A) SNPs rapidly amplified the MT plasmids within 20–30 min, whereas the same primer sets failed to amplify the WT plasmids (complete suppression of the mismatch amplification) by 60 min. WT primer sets designed to target the WT genotypes failed to amplify the MT plasmids. Each assay was run in duplicate and all negative control reactions included in the experiments showed no amplification within 60 min. These results confirmed that the SmartAmp2 assays were optimized, as they were able to accurately discriminate the full match amplification from the mismatch with complete suppression of mismatch amplification. The assay optimization with plasmids for *T*. *trichiura* is shown in (Fig 4). A similar amplification profile was obtained with plasmids for *A*. *lumbricoides*.

### SmartAmp2 assay sensitivity and specificity

**Fig 5:**
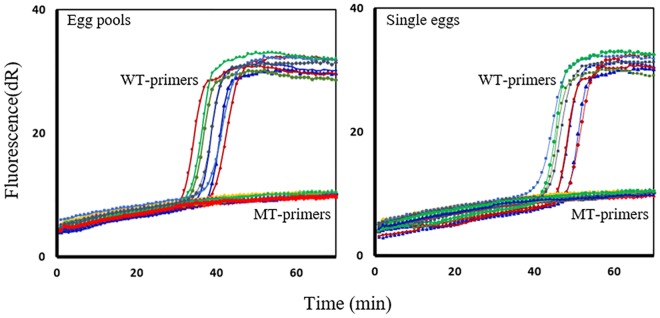
SmartAmp2 assay evaluation with pools and single eggs of *Ascaris lumbricoides* and *Trichuris trichiura*. Wild-type (WT)-specific primer amplification of gDNA from egg pools (30–40 min) with complete suppression of amplification using the mutant-type (MT)-primer sets (left). WT-specific primer amplification of gDNA from single eggs (40–50 min) (right) with complete suppression of amplification with the MT-primer sets. Negative controls (no template control) were included in each run. Both graphs show replicated runs for either *A*. *lumbricoides* and *T*. *trichiura* egg pools or single eggs, respectively. Multiple assays (different colors) are shown in each plot showing reproducibility of the assay to distinguish between WT and MT DNA. dR, difference of relative fluorescence unit.

*A*. *lumbricoides* and *T*. *trichiura*-specific SmartAmp2 primers were designed based on β-tubulin sequence alignment of *A*. *lumbricoides*, *T*. *trichiura*, and *N*. *americanus*. The specificity of each SNP-detection assay was tested on MT plasmids (F167Y, F200Y or E198A) and no amplification was detected from the WT or the MT genotype of non-target sequences. This indicates that the SmartAmp2 assay has a high level of specificity for detecting only SNPs of a target sequence and inhibiting amplification from non-target sequences. To test reproducibility and the sensitivity, each assay was applied to analyze egg pools (10–50 eggs/pool) and single eggs for *A*. *lumbricoides* and *T*. *trichiura*. In the SmartAmp2 assays, the WT-primer set amplified the DNA target from egg pools within 30–40 min, and from single eggs within 40–50 min, but no amplification was observed with the MT-primer set or the negative controls. Positive controls (adult gDNA) were always included and showed full match amplification with only the WT-primer sets (Fig 5).

### Genotyping of *T*. *trichiura* field samples

**Fig 6:**
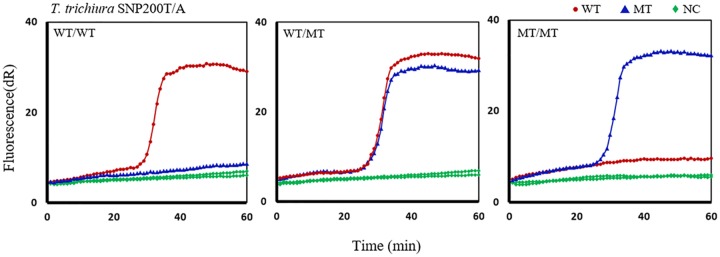
SmartAmp2 genotyping results of *Trichuris trichiura* samples at codon 200. SmartAmp2 amplification of F200Y (T**T**C>T**A**C) using wild type (WT) and mutant type (MT)-primer sets. Left, center, and right panels show assay results for homozygous WT (WT/WT), mixed (WT/MT), and homozygous MT (MT/MT) pooled samples, respectively. dR, difference of relative fluorescence unit.

**Fig 7:**
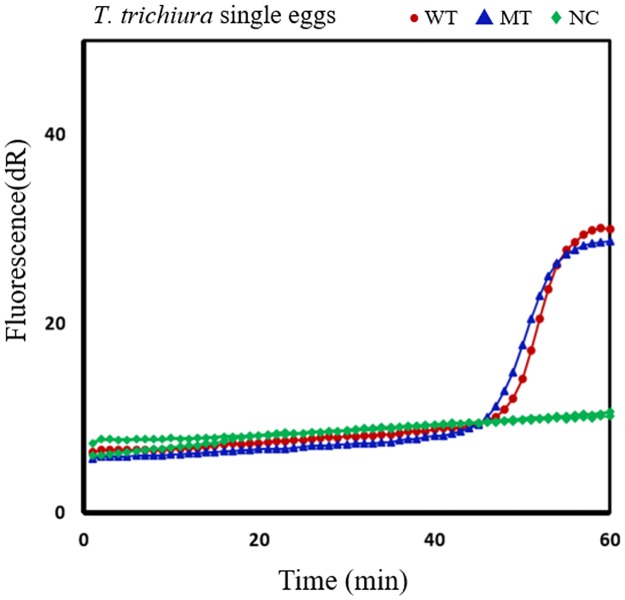
SmartAmp2 genotyping results of *Trichuris trichiura* samples at codon 200. SmartAmp2 amplification of F200Y (T**T**C/T**A**C) with wildtype (WT) and mutant type (MT)-primer sets. The results showed heterozygous (WT/MT) genotypes in *Trichuris* single egg DNA. dR, difference of relative fluorescence unit.

Twenty-five of 40 field samples examined under the microscope, collected from children in Haiti, had *T*. *trichiura* eggs. From each sample, pools of 10 eggs were placed in a PCR tube, and DNA was extracted as described in the Methods. SmartAmp2 assays were fully optimized to target F200Y (T**T**C>T**A**C) and E198A (G**A**A>G**C**A) SNPs in *T*. *trichiura* and were able to detect the presence or absence of WT and MT genotypes within 30 min after incubation at 60°C. No amplification was observed from negative controls. Positive controls (adult gDNA) were always included and showed full match amplification with only the WT-primer sets, as expected. For codon position 198, the WT-primer set allowed amplification of the DNA target within 30 min, but no amplification was observed with the MT-primer set. This indicates that no polymorphism was found at codon position 198 in the *T*. *trichiura* eggs analyzed. However, a polymorphism F200Y (T**T**C>T**A**C) was identified and the MT-primer set allowed amplification of the DNA target within 30 min (full match amplification). The 200SNP detection primer set recognized the F200Y (T**T**C>T**A**C) and discriminated homozygous 200T/T (WT), mixed 200 T/A (WT/MT), and homozygous 200A/A (MT) in *Trichuris* egg pools (Fig 6). To verify the genotyping results for codon 200 obtained with *Trichuris* egg pools, all experiments were repeated twice and performed in duplicate from the same samples and assays were always run with positive and negative controls. Additionally, single eggs from the same samples that had MT genotypes were analysed and the results confirmed the presence of the same MT genotypes obtained with egg pools. By analyzing single eggs, samples that had mixed WT and MT alleles were verified to have heterozygous genotypes at F200Y (T**T**C/T**A**C) SNP (Fig 7).

### Genotyping of *A*. *lumbricoides* field samples

**Fig 8:**
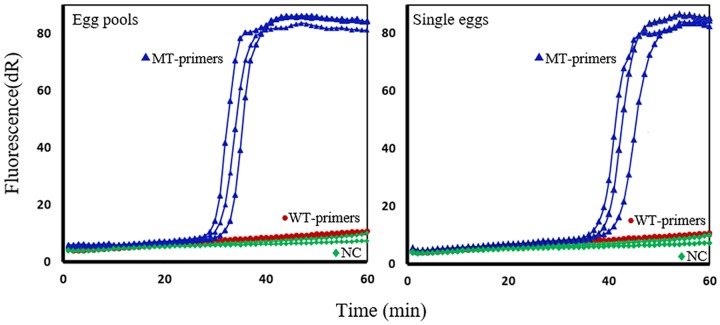
SmartAmp2 genotyping results of *Ascaris lumbricoides* samples at codon 167. SmartAmp2 amplification of β-tubulin isotype 1 gene targeting the F167Y (T**T**C>T**A**C) SNP using the wild-type (WT) (red circle) and mutant-type (MT) (blue triangle)-primer sets. The MT-primer set allowed the full match amplification from egg pools (left) and single eggs (right). No amplification was observed from the WT-primer set or the negative control (green diamond). dR, difference of relative fluorescence unit.

Thirty-four fecal samples collected from children in Panama were examined under the microscope and 20 samples had *A*. *lumbricoides* eggs. For each individual sample, pools of 10 eggs were placed in a PCR tube and DNA was extracted as described in the Methods. SmartAmp2 assays were fully optimized to target the F167Y (T**T**C>T**A**C), F200Y (T**T**C>T**A**C) and E198A (G**A**A>G**C**A) SNPs in *A*. *lumbricoides* and were able to detect the presence or absence of WT and MT genotypes within 30 min. In the Panama samples, the WT-primer sets allowed full match amplification targeting the three codon positions, but no amplification was observed with the MT primer sets. This indicated that there was no polymorphism in these samples at codons 167, 198, or 200. However, in *A*. *lumbricoides* samples obtained from Haiti [21] (4 pools of 10 eggs each), the MT primer set allowed full match amplification within 30 min targeting the F167Y (T**T**C>T**A**C) SNP, but no amplification was observed with the WT-primer set. To verify the results, 40 individual eggs were tested in SmartAmp2 assays and were found to have the homozygous MT alleles at codon 167 (Fig 8). Genotyping results were confirmed by both conventional Sanger sequencing and pyrosequencing.

### SmartAmp2 polymerase tolerance to fecal inhibitors

**Fig 9:**
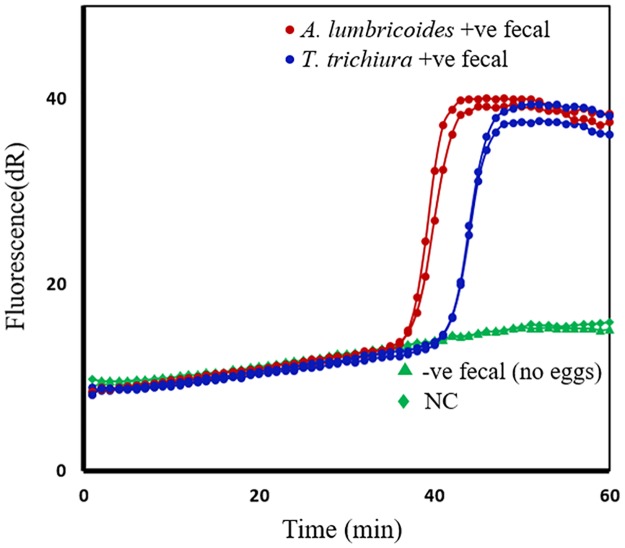
Evaluation of polymerase tolerance in fecal samples spiked with *Trichuris trichiura* or *Ascaris lumbricoides* eggs. SmartAmp2 amplification using the wildtype (WT)-primer set on fecal samples spiked with *A*. *lumbricoides* eggs (red circle), *T*. *trichiura* eggs (blue circle) and negative fecal samples (green triangle) without eggs. Negative controls (no template) were included in each run (green diamond). dR, difference of relative fluorescence unit.

To assess the tolerance of *Aac* polymerase to fecal inhibitors, fecal samples (spiked with eggs) and negative fecal samples were assessed in triplicate in the SmartAmp2 assay. High amplification efficiency was achieved when DNA samples were diluted 4-fold. Full-match amplification was obtained only from positive fecal samples using the WT-primer set within 40–45 min while the negative fecal samples and the negative controls remained at the base line for at least 90 min (Fig 9). This indicates that the *Aac* polymerase is highly tolerant (resistant) to fecal inhibitors even when crude sample preparations were used.

## Discussion

BZ-resistance is a serious problem in veterinary parasites, and the intensive use of these drugs in MDA against human parasites raises concern that resistance may be selected in human STH. The development of rapid and sensitive methods for the detection of resistance-associated SNPs is needed for monitoring the presence and extent of BZ-resistant nematodes. Isothermal diagnostic methods have proven rapidity, sensitivity and specificity for the diagnosis of many parasitic, viral, bacterial and fungal infections [38, 39]. Among these methods, SmartAmp2 is a unique DNA amplification method for rapid detection of genetic polymorphisms under isothermal conditions in a single step, which eliminates the need for PCR amplification, a thermocycler or electrophoresis [31]. This technique uses asymmetrical primer design and the *Aac* DNA polymerase. This polymerase is highly resistant to cellular contaminants in clinical samples and works on crude sample preparations after a simple heating step to degrade RNA and denature proteins [29, 33]. Moreover, inclusion of the *Thermus aquaticus* MutS (*Taq* MutS) enzyme in the isothermal assay completely suppresses the exponential back-ground amplification (mismatch amplification), resulting in high specificity in distinguishing a specific target sequence based on one nucleotide difference [25]. Thus SmartAmp2 has unique advantages over PCR-based methods such as diagnostic real time PCR and pyrosequencing.

In this study, we developed a new SNP genotyping assay based on the SmartAmp2 method for monitoring β-tubulin polymorphisms. Optimal primer sets were selected and optimized specifically to target the F200Y (T**T**C>T**A**C) and E198A (G**A**A>G**C**A) SNPs in *T*. *trichiura* and *A*. *lumbricoides*, in addition to the F167Y (T**T**C>T**A**C) SNP in *A*. *lumbricoides*, SNP-detection primer sets efficiently and rapidly discriminated MT and WT genotypes using plasmids as DNA templates. The SmartAmp2 assay has high specificity for detecting only SNPs of a specific target sequence. A unique advantage of SmartAmp2 is the ability to genotype a SNP in highly homologous regions without cross-amplification of closely related genes. However, this requires intensive primer modification and optimization, particularly when targeting a highly conserved gene such as β-tubulin.

The assays showed high reproducibility and sensitivity for detecting genomic DNA from single egg DNA within 40–50 min in a single amplification and detection step. To detect amplified fragment from samples with low DNA concentrations, using a PCR based method, a nested PCR consisting of two consecutive rounds of amplification using the same primers, followed by gel electrophoresis is required [21]. This multistep technique is time consuming and also increases the risk of contamination as a result of manipulating the PCR product.

SmartAmp2 assays were developed to identify the F200Y (T**T**C>T**A**C) SNP in *T*. *trichiura*. The MT-detection primer set detected MT homozygous and mixed genotypes in *T*. *trichiura* samples from Haiti. This finding was consistent with previous studies in which the SNP 200 was identified in *T*. *trichiura* samples from Kenya [19] and other Haitian samples [21]. In *A*. *lumbricoides*, SmartAmp2 assays were developed to target the F167Y (T**T**C>T**A**C), F200Y (T**T**C>T**A**C) and E198A (G**A**A>G**C**A) SNPs and the MT-detection primer set detected the MT genotype F167Y (TTC>T**A**C) in *A*. *lumbricoides* samples from Haiti. This genotyping result was consistent with previous analyses on *Ascaris* samples from Haiti, Panama and Kenya using pyrosequencing and conventional sequencing [21]. No polymorphism was identified at codon 167 in the *A*. *lumbricoides* samples from Panama. Previous analysis of other *A*. *lumbricoides* samples from Panama found polymorphism at codon 167 [21].

Furthermore, fecal samples spiked with STH eggs were processed in the SmartAmp2 assay and the results showed the high tolerance of the *Aac* polymerase to fecal inhibitors in crude sample preparations. The *Bst 2*.*0* DNA polymerase was initially used and was also found to exhibit high tolerance to fecal inhibitors. However, more suppression of mismatch amplification was observed with the *Aac* polymerase.

We were not successful in the optimization of a SNP detection assay to identify a F167Y (TTT>T**A**T) SNP in *T*. *trichiura*. As optimal primer design mainly depends on the target sequence, we were not able to optimize a set that could specifically distinguish the MT genotype from the WT genotype.

Compared with PCR-based methods, the SmartAmp2 assay is considered relatively inexpensive; the main costs are for the *Taq* MutS and the polymerase. Primers used in this study were not HPLC purified. Additionally, reducing the reaction mixture to 10 μl and using in-house prepared buffers and reagents can also reduce costs. Our data were generated on a RT-PCR system to follow the formation of double stranded DNA in real-time, using SYBR green. However, end point detection system for monitoring fluorescence that would allow high-throughput analysis of samples in a 96-well microplate format could be employed. Other approaches for visualizing the formation of DNA residues could be applied using fluorescence dyes that allow colorimetric screening of the results by the naked eye.

A limitation of our study is that the assay can detect only one SNP at a time, but SmartAmp2 offers simplicity of sample and reaction preparation, rapidity in detection and the convenience of isothermal amplification, which are significant advantages over other genotyping technologies. Additionally, in this study, we simplified the detection of the 198 and 200 SNPs by designing one assay with only a specific discrimination primer for each SNP.

The present study provides evidence that the SmartAmp2 method targeting β-tubulin polymorphisms in STHs allowed direct detection of SNPs of a target DNA sequence in field samples. Additionally, these results indicate that our SNP genotyping assays are rapid, simple, very sensitive and highly specific, which provide unique tools for investigating BZ resistance in STHs. The development of rapid and sensitive tools for detecting resistance-associated alleles would therefore be a key for monitoring the appearance and spread of potential BZ-resistant STHs.
